# The impact of myocardial injury on outcomes in TAVI patients

**DOI:** 10.1007/s00392-024-02585-1

**Published:** 2024-12-11

**Authors:** Thorald Stolte, Pedro Lopez-Ayala, Jakob Reichl, Anna Pfenniger, Giampiero Allegra, Gregor Leibundgut, Christoph Kaiser, Jasper Boeddinghaus, Felix Mahfoud, Christian Mueller, Thomas Nestelberger

**Affiliations:** 1 Department of Cardiology and Cardiovascular Research Institute Basel (CRIB), University Hospital Basel, University of Basel, Petersgraben 4, CH-4031 Basel, Switzerland; 2 Department of Health Sciences and Technology, Swiss Federal Institute of Technology, Zurich, Switzerland; 3 Department of Cardiac Surgery, University Hospital Basel, University of Basel, Basel, Switzerland

**Keywords:** Transcatheter aortic valve implantation, High-sensitivity cardiac troponin T, Peri-procedural myocardial injury

## Abstract

**Background:**

Peri-procedural myocardial injury (PPMI) has been commonly reported after transcatheter aortic valve implantation (TAVI) and may have a potential impact on outcomes. The recent update to the Valve Academic Research Consortium (VARC)-3 criteria for PPMI warrants a comparison with the preceding VARC-2 criteria to understand its implications on patient outcomes.

**Aims:**

To assess the prognostic significance of PPMI as defined by VARC-3 versus VARC-2 in TAVI patients and evaluate the predictive value of high-sensitivity cardiac troponin T (hs-cTnT) for adverse outcomes within 1 year post-TAVI.

**Methods:**

Consecutive patients undergoing TAVI in a tertiary university hospital between December 2011 and June 2023, with hs-cTnT concentrations pre- and post-procedurally, were enrolled. The primary outcome was all-cause mortality at 1 year. Secondary outcomes were major cardiac adverse events (MACE), defined as a composite end point including all-cause mortality, unplanned reintervention, stroke, myocardial infarction, or major bleeding at 30 days and 1 year.

**Results:**

Of 653 patients, 535 (82%) had elevated baseline serum hs-cTnT. It was a significant predictor of 1-year mortality and MACE, whereas post-TAVI hs-cTnT concentrations did not predict outcomes (HR: 1.5, *p* = 0.21 and HR: 0.943, *p* = 0.54). 367 (56%) of all patients met VARC-2 PPMI criteria, while only 24 (3.7%) met VARC-3 criteria. Patients meeting VARC-3 criteria had significantly more comorbidities and higher 1-year mortality (25% vs. 9%; *p* = 0.0047). VARC-2 criteria did not predict higher mortality (9% vs. 9%; *p* = 0.69).

**Conclusions:**

Baseline hs-cTnT concentrations strongly predicted 1-year mortality and MACE, while post-procedure levels did not. VARC-3 criteria provided better prognostic discrimination than VARC-2.

**Supplementary Information:**

The online version contains supplementary material available at 10.1007/s00392-024-02585-1.

## Introduction

Transcatheter aortic valve implantation (TAVI) has become a widely used, efficient, and safe alternative to surgical aortic valve replacement (SAVR), based on clinical trials suggesting comparable efficacy and safety profiles among older patients irrespective of risk categories. [1–5] Peri-procedural myocardial injury (PPMI) is a possible complication after TAVI. While several studies have suggested a relevant impact on outcomes, [6–8] others have shown no clear association [9, 10]. Sources of PPMI include global myocardial ischemia caused by rapid pacing, coronary obstruction, micro-embolization of aortic valve debris into the coronary arteries, myocardial tissue compression from the expansion of the valve, and access related trauma (e.g., transapical procedures) [11–14].

The Valve Academic Research Consortium (VARC)−2 defined PPMI solely based on biomarkers, including high-sensitivity cardiac troponin T (hs-cTnT) with an elevation over 15 times the upper reference limit (URL), irrespective of ischemic symptoms or signs together with clinical and/or ECG findings [15]. Recently, the VARC-3 raised the threshold for hs-cTnT to 70 times the URL, or 35 times the URL together with clinical and/or ECG findings [16]. To date, only one study has used VARC-3 criteria to determine the incidence and outcomes of patients with PPMI and compared it to VARC-2-defined PPMI, showing that VARC-2 had no prognostic impact on mortality while VARC-3 was associated with a higher risk of mortality at 30-day and 1-year [17]. However, a critical gap persists in the incorporation of baseline hs-cTnT measurements into the calculation of PPMI as defined by both VARC-2 and VARC-3. Additionally, there is a lack of exploration into the prognostic significance of VARC-3-defined PPMI in comparison to absolute hs-cTnT values at baseline and post-procedurally, as well as relative increases, which has not been previously investigated.

The present study aims to determine the incidence and prognostic impact of VARC-3-defined PPMI following TAVI in a large prospective registry in patients with hs-cTnT measurements before and after the procedure. Additionally, the study explores the prognostic significance of VARC-3-defined PPMI in comparison to absolute hs-cTnT values at baseline and post-procedurally, along with relative increases.

## Methods

### Study design and patient cohort

Consecutive patients undergoing TAVI at the University Hospital Basel (USB), Switzerland, were included in a prospective national database, as part of the Swiss TAVI registry, mandated by the Swiss health authorities (NCT01368250). The Swiss TAVI registry has been approved by the local ethics committee and the institutional review board. All patients provided written informed consent for study participation. Prior results from the Swiss TAVI registry have been reported elsewhere [18–20]. The present retrospective analysis of a prospective study included all patients who underwent TAVI between December 2011 and June 2023 and for whom hs-cTnT measurements within 30 days pre- and 72 h post-procedurally were available.

### Data collection and clinical end points

Patient-related data including baseline characteristics, procedural, and follow-up information was prospectively collected and recorded in a web-based database. Clinical follow-up data was obtained through standardized interviews, documentation from referring physicians, and hospital discharge summaries. All adverse events were systematically collected and adjudicated by a dedicated clinical event committee based on the VARC-3 definitions, which were adjudicated based on detailed documentation of reported end points [16].

PPMI was defined according to VARC-2 and VARC-3 separately. VARC-2-defined PPMI was satisfied if peak hs-cTnT exceeded 15 times the assay-specific uniform upper reference limit (URL) 99th percentile (14 ng/L) in patients with normal baseline hs-cTnT, or a further increase in at least 50% in patients with elevated baseline hs-cTnT within 72 h post-TAVI. VARC-3-defined PPMI was satisfied if hs-cTnT increased > 70 times above the URL or > 35 times in the presence of left bundle branch block. Clinical end points were defined as the incidence of troponin elevation in patients undergoing TAVI before or after procedure, including absolute and relative changes.

The primary outcome was all-cause mortality within 1 year. The secondary end points were major adverse cardiac events (MACE), defined as a composite end point including all-cause mortality, unplanned reintervention, stroke, myocardial infarction, or major bleeding (BARC III or V) at 30 days and at 1 year.

### Laboratory testing

Hs-cTnT was measured from heparin plasma samples as part of clinical routine blood sampling (Elecsys 2010 hs-cTnT, Roche Diagnostics). The 99th percentile of the assay is 14 ng/L with a coefficient of variation of 10% at 13 ng/L. The limit of blank and limit of detection were determined to be 3 and 5 ng/L, respectively [21]. For the current analysis, all available concentrations were measured within 30 days prior and 72 h after TAVI. If multiple measurements were obtained within the designated time frame, the measurement closest in time to the procedure was selected for analysis.

### Statistical analysis

Categorical variables are reported as count (percentage), and continuous variables as median (interquartile range [IQR]). Comparisons between continuous variables were performed using Mann–Whitney *U* test. Categorical variables were compared using Pearson’s Chi-squared test or Fisher’s exact test, as appropriate. Spaghetti plots and boxplots with whiskers were utilized to illustrate disparities in troponin values before and after the intervention. Survival analysis was carried out using the Kaplan–Meier method and differences in survival tested with the log-rank test. Multivariable Cox proportional hazards models with adjustments for age, estimated glomerular filtration rate (eGFR) valve type and procedural access were used to evaluate the association of VARC-2, VARC-3-defined PPMI, as well as absolute hs-cTnT values pre- and post-interventionally with 1-year all-cause mortality and MACE, respectively. A partial (conditional) effects plots was created to assess the relationship of a baseline hs-cTnT to the probability of death and MACE at 1 year post-interventionally. A *p* value < 0.05 was considered statistically significant. All statistical analyses were performed using R 4.2.3 (R Foundation for Statistical Computing, Vienna, Austria).

## Results

### Patient characteristics

**Fig. 1:**
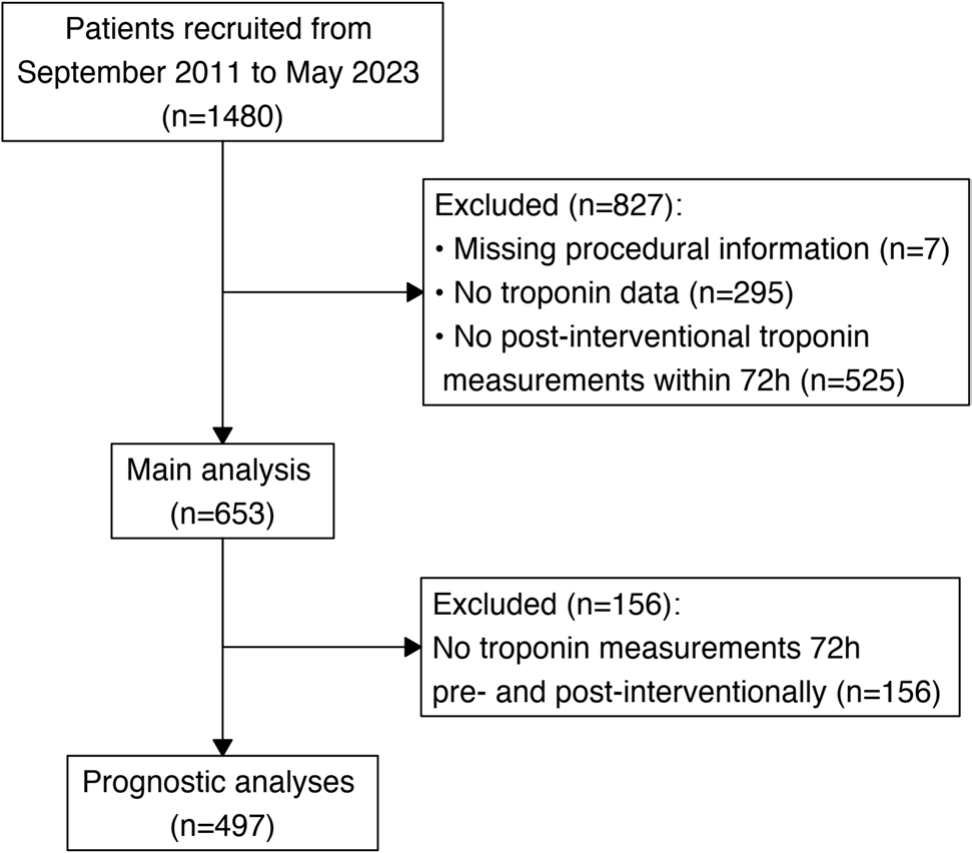
Patient flowchart

Among 1480 patients who underwent TAVI between September 2011 and May 2023, hs-cTnT measurements taken within 30 days before and 72 h after the procedure were available for 653 patients (44%) (Fig. 1). There was no difference between patients with and without available hs-cTnT-levels (Supplementary Table 1). The median age was 82.5 [79–86] years, 46% were females, 60% had known coronary artery disease (CAD) with a median left ventricular ejection fraction (LVEF) of 57%, and an eGFR of 52 [38–68] ml/min/1.73 m^2^ (Table 1).

**Table 1:** Patient baseline characteristics according to VARC-3-defined PPMI

Variable	Overall, *N* = 653^*1*^	PPMI, *N* = 24^*1*^	Non-PPMI, *N* = 629^*1*^	*p* value^*2*^
Sex				0.2
Female	301 (46%)	8 (33%)	293 (47%)	
Age [years]	82.5 (78.9, 85.9)	81.4 (78.8, 87.5)	82.6 (78.9, 85.9)	> 0.9
BMI [kg/m^2^]	26.4 (23.4, 29.4)	24.2 (22.9, 27.8)	26.4 (23.5, 29.4)	0.12
Euro SCORE II	2.3 (1.4, 4.7)	5.4 (3.2, 8.8)	2.2 (1.3, 4.5)	< 0.001
STS calculated risk of mortality	3.4 (2.2, 5.7)	6.1 (3.4, 8.9)	3.3 (2.2, 5.7)	0.002
Comorbidities				
Diabetes	197 (30%)	6 (25%)	191 (30%)	0.6
Dyslipidemia	404 (62%)	13 (54%)	391 (62%)	0.4
Hypertension	543 (83%)	19 (79%)	524 (83%)	0.6
Peripheral artery disease	143 (22%)	9 (38%)	134 (21%)	0.06
Coronary artery disease	389 (60%)	18 (75%)	371 (59%)	0.12
Prior myocardial infarction	116 (18%)	7 (29%)	109 (17%)	0.2
eGFR (Cockroft–Gault) [mL/m^2^]	52 (38, 68)	42 (20, 50)	53 (38, 68)	< 0.001
Hemoglobin [g/L]	127 (114, 138)	124 (104, 135)	127 (114, 138)	0.2
Previous interventions				
Prior heart surgery	78 (12%)	3 (13%)	75 (12%)	> 0.9
Surgical aortic valve replacement	18 (2.8%)	0 (0%)	18 (2.9%)	> 0.9
Coronary artery bypass grafting	67 (10%)	3 (13%)	64 (10%)	0.7
Prior pacemaker	60 (9.2%)	0 (0%)	60 (9.5%)	0.2
Percutaneous coronary intervention	243 (37%)	12 (50%)	231 (37%)	0.2
ECG characteristics				
Any AV block	100 (19%)	4 (18%)	96 (19%)	> 0.9
Right or left bundle branch block				0.012
LBBB	74 (13%)	8 (33%)	66 (12%)	
RBBB	64 (11%)	3 (13%)	61 (11%)	
No	421 (75%)	13 (54%)	408 (76%)	
Rhythm				0.7
Sinus	408 (71%)	19 (79%)	389 (70%)	
Atrial fibrillation	131 (23%)	5 (21%)	126 (23%)	
Paced_rhythm	33 (5.7%)	0 (0%)	33 (6.0%)	
Other	5 (0.9%)	0 (0%)	5 (0.9%)	
Echocardiography parameters				
LVEF [%]	57 (45, 63)	53 (43, 61)	57 (45, 63)	0.3
Aortic valve mean gradient [mmHg]	44 (36, 52)	47 (38, 54)	44 (35, 52)	0.7
Aortic valve peak gradient [mmHg]	66 (47, 79)	76 (50, 88)	66 (47, 78)	0.13
Aortic valve area [cm^2^]	0.8 (0.60, 0.90)	0.75 (0.60, 0.97)	0.8 (0.60, 0.90)	> 0.9

^*1*^*N* (%); median (IQR)

^*2*^Fisher’s exact test; Wilcoxon rank sum test

### Peri-procedural myocardial injury

**Fig. 2:**
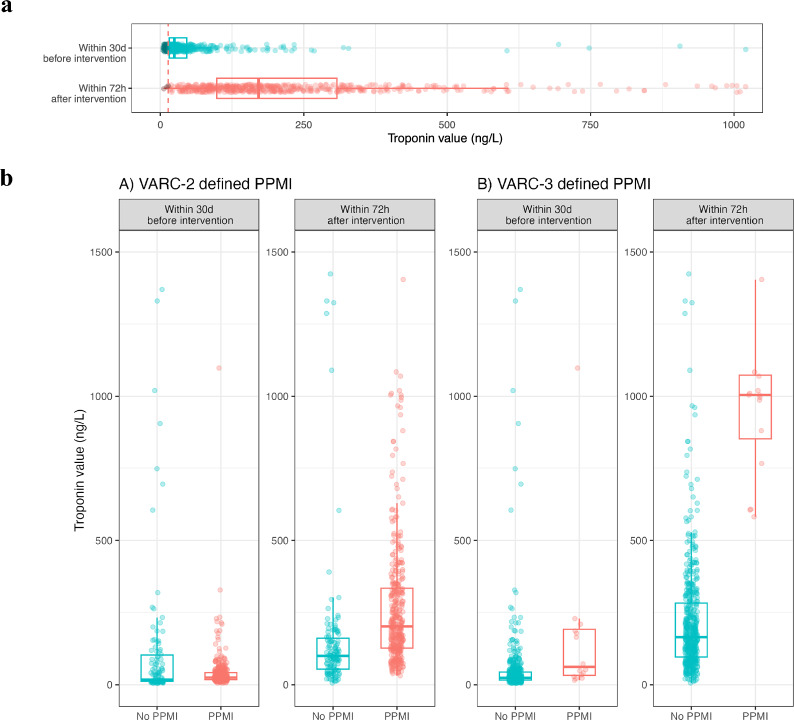
**a** Elevation of troponin from before to after interventions. The red dashed line represents the upper reference limit cutoff of 14 ng/L. **b** Comparison of high-sensitivity troponin T elevations for VARC-2 & 3 defined peri-procedural myocardial injury (PPMI)

Baseline serum hs-cTnT-levels were elevated above the 99th percentile in 82% of all non-PPMI and 100% of PPMI patients before TAVI, increasing to over 99% in all patients after the procedure (Fig. 2a). In total, 88% of patients exhibited an increase of over 20% from pre- to post-TAVI measurements.

VARC-2-defined PPMI was present in 367 (56%) patients, whereas VARC-3-defined PPMI was diagnosed in 24 patients (3.7%). Patients with VARC-3-defined PPMI exhibited a median 73-fold increase in post-TAVI hs-cTnT levels, while those with VARC-2-defined PPMI displayed a 14-fold increase (Fig. 2b).

Patients with VARC-3-defined PPMI had a significantly higher risk of mortality according to Euro SCORE II and STS Calculated risk of mortality assessment (5.4% [IQR 3.2, 8.8] and 6.1% [IQR 3.4, 8.9] vs. 2.2% [IQR 1.3, 4.5] and 3.3% [IQR 2.2, 5.7]; *p* < 0.001 and *p* = 0.002) and lower eGFR (42 [IQR 20, 50] vs 53 ml/min/1.73 m^2^ [38, 68]; *p* < 0.001). There were no differences in terms of age, pre-existing conditions, or previous interventions between the groups (Table 1). PPMI patients were significantly more likely to present with left bundle branch blocks (LBBB) than non-PPMI patients (33% vs 12%; *p* = 0.012) but showed no other differences in conduction disturbances. Additionally, there were no differences in baseline echocardiographic parameters between the two groups (Table 1).

### Procedural characteristics

Device type and size were similar in patients with and without VARC-3-defined PPMI (*p* = 0.5 and *p* = 0.7). Overall, 516 patients (79%) underwent extrathoracic vascular access for TAVI (69% transfemoral) and 137 (21%) intrathoracic access. Patients who underwent trans-apical or direct aortic access had a significantly higher median increase in hs-cTnT than those who underwent femoral or subclavian access (26-fold vs 11-fold, *p* < 0.001) (Supplemental Fig. 1). VARC-3-defined PPMI was significantly more prevalent in patients with intrathoracic access, accounting for over half of all access procedures (54% vs 20%; *p* < 0.001).

Furthermore, implantation duration and hospitalization were significantly longer in patients with PPMI (81 min [80, 118] vs 65 min [52, 85] and 13.9 days [8.6, 19.9] vs. 5.9 days [130, 217]; *p* = 0.035 and *p* < 0.001). Peri-procedural resuscitation occurred in 1.6% of cases, and in-hospital death occurred in 3.3% of patients. There were no differences in the incidence of cerebrovascular or cardiovascular complications between the groups. However, PPMI patients underwent significantly more repeated unplanned interventions, including balloon valvuloplasty, percutaneous coronary interventions (PCI), surgical aortic valve replacement, and valve-in-valve procedures (8.3% vs. 1.0%; *p* = 0.032) (Table 2).

**Table 2:** Patient procedural characteristics and procedural complications between patients with and without VARC-3-defined PPMI

Variable	Overall, *N* = 653^*1*^	PPMI, *N* = 24^*1*^	Non-PPMI, *N* = 629^*1*^	*p* value^*2*^
Intervention characteristics				
Implanted device type				0.5
balloon-expandable	213 (33%)	10 (42%)	203 (32%)	
Mechanical and self-expandable	439 (67%)	14 (58%)	425 (68%)	
Implanted device size [mm]				0.7
≤ 25	222 (34%)	9 (38%)	213 (34%)	
> 25	429 (66%)	15 (63%)	414 (66%)	
Access site				< 0.001
Extrathoracic	516 (79%)	11 (46%)	505 (80%)	
Intrathoracic	137 (21%)	13 (54%)	124 (20%)	
Implantation duration [min]	65 (52, 85)	81 (60, 118)	65 (52, 85)	0.037
Hospitalization [days]	6.8 (3.9, 8.9)	13.9 (8.6, 19.9)	5.9 (3.9, 8.8)	< 0.001
Contrast volume [ml]	165 (129, 217)	133 (120, 220)	165 (130, 217)	0.3
Serious adverse events				
Peri-procedural death	8 (1.2%)	0 (0%)	8 (1.3%)	> 0.9
Peri-procedural resuscitation	10 (1.6%)	1 (4.3%)	9 (1.5%)	0.3
In-hospital death	6 (3.3%)	2 (15%)	4 (2.4%)	0.060
Repeated unplanned intervention	8 (1.2%)	2 (8.3%)	6 (1.0%)	0.032
Acute coronary occlusion	2 (0.3%)	0 (0%)	2 (0.3%)	> 0.9
Cerebrovascular events	50 (9%)	4(17%)	46 (7%)	0.09
Transient ischemic attack	19 (2.9%)	2 (8.3%)	17 (2.7%)	
Stroke	31 (4.7%)	2 (8.3%)	29 (4.6%)	
Permanent pacemaker implantation	106 (16%)	6 (25%)	100 (16%)	0.2
Higher-grade AV block	78 (12%)	4 (17%)	74 (12%)	
Other	28 (4.3%)	2 (8.3%)	26 (4.1%)	
Myocardial infarction	17 (3%)	1 (4.2%)	16 (3%)	0.5
Peri-procedural	11 (1.7%)	1 (4.2%)	10 (1.6%)	
Spontaneous	6 (0.9%)	0 (0%)	6 (1.0%)	
Vascular complication	96 (15%)	2 (8%)	94 (15%)	0.6
Major	28 (4.3%)	1 (4.2%)	27 (4.3%)	
Minor	68 (10%)	1 (4.2%)	67 (11%)	

^*1*^*N* (%); median (IQR)

^*2*^Fisher’s exact test; Wilcoxon rank sum test

### Primary and secondary outcomes

**Fig. 3:**
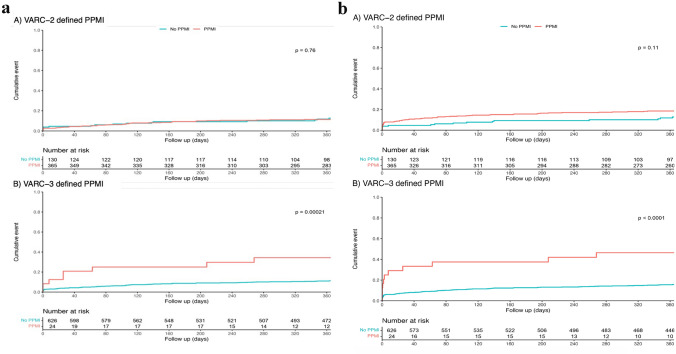
**a** Comparison of Kaplan–Meier 1-year mortality between VARC-2- and VARC-3 defined PPMI versus non-PPMI. **b** Comparison of Kaplan–Meier 1-year MACE-incidence between VARC-2- and VARC-3-defined PPMI versus non-PPMI

Forty-four (9%) of the 495 patients reached the primary end point of all-cause mortality within 1 year post-TAVI. Patients with VARC-3-defined PPMI had a significantly higher rate of death at 1 year than those without (6/24 (25%) vs 59/626 (9%), *p* = 0.0047), which did not remain significant after adjustment for age and eGFR (HR: 1.97 (0.94–4.13), *p* = 0.13). There was no difference between patients with VARC-2-defined PPMI and those without (33/365 (9%) vs 12/130 (9%), *p* = 0.69; adjusted HR: 1.21 (0.7–2.07), *p* = 0.57) (Fig. 3a).

The incidence of MACE at 30 days was 8.6% in the overall population and was significantly higher in patients with VARC-3-defined PPMI (8/24 (33.3%) vs 48/626 (7.6%), *p* < 0.0001), which was independent of age and eGFR (adjusted HR: 3.02 (1.73–5.26), *p* < 0.001). No significant difference was observed when comparing patients with VARC-2-defined PPMI to those without (37/365 (10%) vs 6/130 (4.6%), *p* = 0.062; adjusted HR: 0.66 (0.42–1.04), *p* = 0.13). At 1 year, the incidence of MACE was 16% in the overall population and exhibited a significant difference in patients with VARC-3-defined PPMI (11/24 (46%) vs 94/626 (15%), *p* < 0.0001; adjusted HR: 3.0, *p* = 0.001), while no significant difference was found between patients with VARC-2-defined PPMI and those without (67/365 (18%) vs 15/130 (12%), *p* = 0.11; adjusted HR: 1.5, *p* = 0.11) (Fig. 3b).

**Fig. 4:**
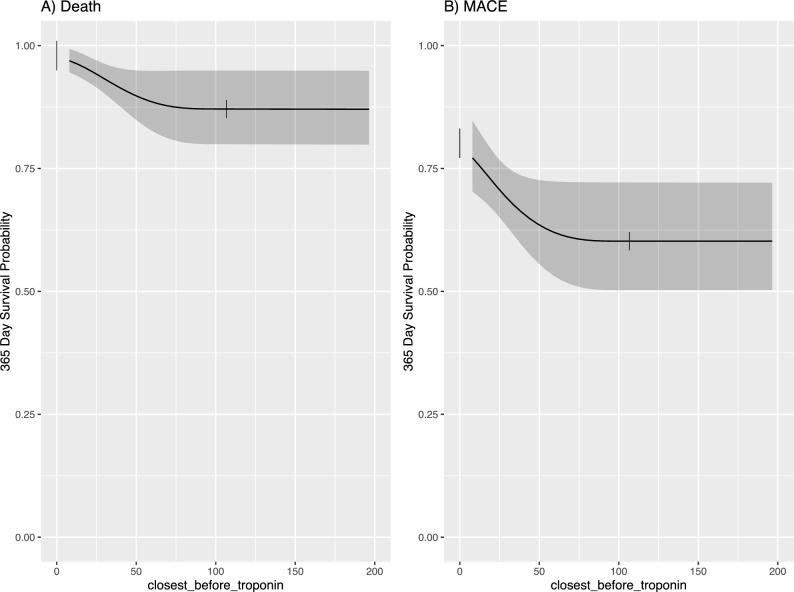
Partial (conditional) effects plots of baseline hs-cTnT for **A** death and **B** major adverse cardiac events (MACE). Y-axis represents the probability of event-free survival for MACE/death at 1 year

Patients with elevated baseline hs-cTnT concentrations showed significantly higher incidences of MACE (HR: 2.2 (1.15–2.60), *p* = 0.0215) and all-cause mortality at 1 year (HR: 2.6 (1.27–4.25), *p* = 0.026), while neither a relative increase of any percentage nor absolute levels of post-TAVI hs-cTnT were predictive of MACE or death at 1 year (percentage increase: HR: 0.93 (0.6–1.42), *p* = 0.48 and HR: 0.72 (0.36–1.44); absolute values post-TAVI: HR: 1.5 (0.23–2.77), *p* = 0.21 and HR: 0.94 (0.79–2.14), *p* = 0.54) (Fig. 4) (see Supplemental Table 2 and Supplemental Table 3 for adjustment for device type and procedural access).

## Discussion

In this study, we report the incidence and outcomes of patients with PPMI after TAVI using both the VARC-2 and VARC-3 definitions over the last 12 years in a tertiary care setting. This cohort is among the largest to date with hs-cTnT level measurements taken before and after the procedure, applying both criteria. We present two major findings.

First, we observed that a majority of patients (82%) undergoing TAVI had elevated levels of hs-cTnT at baseline, consistent with prior reports [22–24]. Elevated hs-cTnT concentrations before the procedure highlight the older age of this patient cohort (median age 82.5 years) and the high prevalence of cardiovascular morbidities, such as coronary artery disease (60%), hypertension (83%), or impaired renal function (62% with eGFR ≤ 60 ml/min/1.73 m^2^). Baseline troponin elevation also correlated with adverse outcomes, including MACE and all-cause mortality at 1 year (HR: 2.2 (1.15–2.60), *p* = 0.0215 and HR: 2.6 (1.27–4.25), *p* = 0.026, respectively), corroborating previous studies [23, 25]. Ferrer-Sistach et al. reported that even a slight elevation in troponin concentration in asymptomatic patients with severe AS correlated with adverse outcomes, highlighting troponin as an independent predictor of adverse outcomes in patients with AS [23]. Our findings align, as nearly all patients had an elevated troponin post-procedure (> 99%), and 88% had at least a 20% relative increase in troponin. Interestingly, troponin levels after TAVI and the amount of increase before/after TAVI were not prognostic of adverse outcomes, aligning with some prior studies but contrasting others [23–25]. This suggest that the nature of troponin release during the procedure does not impact outcomes. Furthermore, we found that intrathoracic vascular access resulted in higher troponin concentrations and PPMI after TAVI compared to extrathoracic vascular access (54% vs 20%; *p* < 0.001). Despite this, the higher troponin levels did not result in higher rates of MACE or all-cause mortality at 1 year (13% vs 8%, *p* = 0.15; 20% vs 15%, *p* = 0.3). This finding is supported by existing literature [8], although some studies have reported better survival rates for transfemoral access [26, 27].

Second, VARC-2-defined PPMI was more common than VARC-3-defined PPMI (56% versus 4%). This finding is mainly based on the higher cutoff values for troponin [17]. VARC-3 criteria offered significantly better discrimination of short- and long-term outcomes. Both unadjusted 1-year mortality and MACE were significantly increased in patients with VARC-3-defined PPMI (*p* < 0.001 for both), whereas VARC-2-defined PPMI offered no distinction between groups (*p* = 0.76 and *p* = 0.11), corroborating the findings of Real et al.[17] The lack of a significant difference regarding death after 1 year between groups after adjustment for age and renal function (HR: 1.97 (0.94–4.13), *p* = 0.13) could have been influenced by the small number of events for VARC-3-defined PPMI. Nevertheless, even though VARC-3 appears to offer improved diagnostic clarity compared to VARC-2 and to better predict adverse outcomes, its specific impact on treatment strategies remains ambiguous. This raises the question of whether the differentiation provided by VARC-3 significantly alters clinical management or improves patient outcomes compared to the simpler approach of focusing on myocardial infarction as a primary diagnostic criterion. Especially type 1 myocardial infarction and some subtypes of type 2 myocardial infarction, such as coronary obstruction, embolism, or spasm, which should also be labeled as type 1 myocardial infarction, have shown to be reliable predictors of outcome. [28] Myocardial infarction either caused during the procedure or thereafter, by e.g., acute coronary occlusion, embolization, or acute plaque rupture/erosion, will result in immediate coronary interventions, rhythm monitoring, and dual antiplatelet therapy, whereas myocardial injury as defined by VARC-2 or VARC-3, highlighting a relevant increase in biomarkers irrespective of the reasons, results in no clear treatment strategy. Addressing these considerations is crucial for optimizing patient care and guiding future research in this field.

### Limitations

This study’s limitations primarily include the low number of events defined by the VARC-3 definition of PPMI, which hindered the ability to conduct a fully adjusted multivariable regression analysis. Additionally, the single-center design and retrospective nature of this analysis pose constraints. We acknowledge that external validation in a second, independent cohort would further strengthen the generalizability of our findings. However, due to the aforementioned limitations, this was not feasible in the present study. Our cohort, however, serves as a validation of previous research addressing the same hypothesis, reinforcing the consistency of the findings. Future studies should aim to replicate these results in additional cohorts to provide even stronger evidence. Despite these limitations, our cohort remains one of the largest to date using an hs-cTnT assay. Furthermore, the absence of significant differences in patient characteristics between those with and without troponin measurements mitigates this limitation to some extent, suggesting a balanced representation across the study population.

In conclusion, our 12-year study offers a comprehensive analysis of the incidence and outcomes of PPMI in TAVI patients, utilizing both VARC-2 and VARC-3 criteria. The high prevalence of elevated baseline hs-cTnT concentrations is indicative of the advanced age and pre-existing cardiovascular conditions in this patient cohort. The VARC-3 criteria have proven to be a more effective tool for outcome discrimination. However, its practical implications in clinical management, as compared to traditional myocardial infarction criteria, require further investigation. Elevated baseline hs-cTnT are strong predictors of mortality and MACE at 1 year, whereas post-TAVI hs-cTnT concentrations were not associated with adverse outcomes. Further research is necessary to validate our findings further and explore the clinical utility of elevated baseline hs-cTnT levels.

### Impact on daily practice

This study underscores the superiority of the VARC-3 criteria over VARC-2 in predicting post-TAVI, particularly highlighting the prognostic value of baseline hs-cTnT levels. It validates the relevance of VARC-3 for enhanced risk assessment, representing a significant advancement in patient care. Nonetheless, the integration of VARC-3 criteria into clinical practice and its impact on treatment strategies, especially in comparison to myocardial infarction diagnostics, warrants further research.

## Supplementary Information

Below is the link to the electronic supplementary material.

Supplementary file1 (DOCX 366 KB)

## Data Availability

The data that support the findings of this study are available from the corresponding author upon reasonable request.

## Abbreviations

AS: Aortic stenosis
ECG: Electrocardiogram
hs-cTnT: High-sensitivity cardiac troponin T
IQR: Interquartile range
LBBB: Left bundle branch block
LVEF: Left ventricular ejection fraction
PPMI: Peri-procedural myocardial injury
TAVI: Transcatheter aortic valve implantation
URL: Upper reference limit
VARC: Valve Academic Research Consortium

## References

[1] Mack MJ, Leon MB, Thourani VH, Pibarot P, Hahn RT, Genereux P et al (2023) Transcatheter aortic-valve replacement in low-risk patients at five years. N Engl J Med 389(21):1949–1960 37874020 10.1056/NEJMoa2307447

[2] Forrest JK, Deeb GM, Yakubov SJ, Gada H, Mumtaz MA, Ramlawi B et al (2023) 4-Year outcomes of patients with aortic stenosis in the evolut low risk trial. J Am Coll Cardiol 82(22):2163–2165 37877907 10.1016/j.jacc.2023.09.813

[3] Jørgensen TH, Thyregod HGH, Ihlemann N, Nissen H, Petursson P, Kjeldsen BJ et al (2021) Eight-year outcomes for patients with aortic valve stenosis at low surgical risk randomized to transcatheter vs. surgical aortic valve replacement. Eur Heart J 42(30):2912–2919 34179981 10.1093/eurheartj/ehab375 PMC8347457

[4] Steffen J, Reißig N, Andreae D, Beckmann M, Haum M, Fischer J et al (2022) TAVI in patients with low-flow low-gradient aortic stenosis-short-term and long-term outcomes. Clin Res Cardiol 111(12):1325–1335 35320407 10.1007/s00392-022-02011-4 PMC9681695

[5] Parikh PB, Mack M, Stone GW, Anker SD, Gilchrist IC, Kalogeropoulos AP et al (2024) Transcatheter aortic valve replacement in heart failure. Eur J Heart Fail 26(2):460–470 38297972 10.1002/ejhf.3151

[6] Rodés-Cabau J, Gutiérrez M, Bagur R, De Larochellière R, Doyle D, Côté M et al (2011) Incidence, predictive factors, and prognostic value of myocardial injury following uncomplicated transcatheter aortic valve implantation. J Am Coll Cardiol 57(20):1988–1999 21565636 10.1016/j.jacc.2010.11.060

[7] Ribeiro HB, Nombela-Franco L, Muñoz-García AJ, Lemos PA, Amat-Santos I, Serra V et al (2015) Predictors and impact of myocardial injury after transcatheter aortic valve replacement. J Am Coll Cardiol 66(19):2075–2088 26541917 10.1016/j.jacc.2015.08.881

[8] Koskinas KC, Stortecky S, Franzone A, O’Sullivan CJ, Praz F, Zuk K et al (2016) Post-procedural troponin elevation and clinical outcomes following transcatheter aortic valve implantation. J Am Heart Assoc 5(2):e002430 26896474 10.1161/JAHA.115.002430 PMC4802442

[9] Sinning JM, Hammerstingl C, Schueler R, Neugebauer A, Keul S, Ghanem A et al (2016) The prognostic value of acute and chronic troponin elevation after transcatheter aortic valve implantation. EuroIntervention 11(13):1522–1529 25671517 10.4244/EIJY15M02_02

[10] Carrabba N, Valenti R, Migliorini A, Vergara R, Parodi G, Antoniucci D (2013) Prognostic value of myocardial injury following transcatheter aortic valve implantation. Am J Cardiol 111(10):1475–1481 23465097 10.1016/j.amjcard.2013.01.301

[11] Kim WK, Liebetrau C, van Linden A, Blumenstein J, Gaede L, Hamm CW et al (2016) Myocardial injury associated with transcatheter aortic valve implantation (TAVI). Clin Res Cardiol Off J Ger Card Soc 105(5):379–387 10.1007/s00392-015-0949-6 26670909

[12] Rahhab Z, Labarre Q, Nijenhuis VJ, El Faquir N, De Biase C, Philippart R et al (2019) Myocardial injury post transcatheter aortic valve implantation comparing mechanically expanded versus self-expandable versus balloon-expandable valves. Struct Heart 3(5):431–437

[13] Nano N, Aytekin A, Ndrepepa G, Seguchi M, Bresha J, Alvarez Covarrubias HA et al (2022) Periprocedural myocardial injury according to optical characteristics of neointima and treatment modality of in-stent restenosis. Clin Res Cardiol 111(7):827–837 35476138 10.1007/s00392-022-02024-z PMC9242953

[14] Buergin N, Lopez-Ayala P, Hirsiger JR, Mueller P, Median D, Glarner N et al (2023) Sex-specific differences in myocardial injury incidence after COVID-19 mRNA-1273 booster vaccination. Eur J Heart Fail 25(10):1871–1881 37470105 10.1002/ejhf.2978

[15] Kappetein AP, Head SJ, Généreux P, Piazza N, van Mieghem NM, Blackstone EH et al (2012) Updated standardized endpoint definitions for transcatheter aortic valve implantation: the valve academic research consortium-2 consensus document. J Am Coll Cardiol 60(15):1438–1454 23036636 10.1016/j.jacc.2012.09.001

[16] VARC-3 WRITING COMMITTEE, Généreux P, Piazza N, Alu MC, Nazif T, Hahn RT et al (2021) Valve academic research consortium 3: updated endpoint definitions for aortic valve clinical research. J Am Coll Cardiol 77(21):2717–2746 33888385 10.1016/j.jacc.2021.02.038

[17] Real C, Avvedimento M, Nuche J, Franzone A, Farjat-Pasos J, Trinh KH et al (2023) Myocardial injury after transcatheter aortic valve replacement according to VARC-3 criteria. JACC Cardiovasc Interv 16(10):1221–1232 37225294 10.1016/j.jcin.2023.03.022

[18] Pilgrim T, Franzone A, Stortecky S, Nietlispach F, Haynes AG, Tueller D et al (2017) Predicting mortality after transcatheter aortic valve replacement: external validation of the transcatheter valve therapy registry model. Circ Cardiovasc Interv 10(11):e005481 29127116 10.1161/CIRCINTERVENTIONS.117.005481

[19] Attinger-Toller A, Ferrari E, Tueller D, Templin C, Muller O, Nietlispach F et al (2021) Age-related outcomes after transcatheter aortic valve replacement: insights from the SwissTAVI registry. JACC Cardiovasc Interv 14(9):952–960 33865734 10.1016/j.jcin.2021.01.042

[20] Stolte T, Boeddinghaus J, Allegra G, Leibundgut G, Reuthebuch O, Kaiser C et al (2023) Incidence and outcomes of valve-in-valve transcatheter aortic valve implantation in failed bioprosthetic valves. J Clin Med 12(18):5868 37762811 10.3390/jcm12185868 PMC10531770

[21] Krintus M, Kozinski M, Boudry P, Capell NE, Köller U, Lackner K et al (2014) European multicenter analytical evaluation of the Abbott ARCHITECT STAT high sensitive troponin I immunoassay. Clin Chem Lab Med 52(11):1657–1665 24897400 10.1515/cclm-2014-0107

[22] Akodad M, Roubille F, Marin G, Lattuca B, Macia JC, Delseny D et al (2020) Myocardial injury after balloon predilatation versus direct transcatheter aortic valve replacement: insights from the DIRECTAVI trial. J Am Heart Assoc 9(24):e018405 33297821 10.1161/JAHA.120.018405 PMC7955361

[23] Schoechlin S, Schulz U, Ruile P, Hein M, Eichenlaub M, Jander N et al (2021) Impact of high-sensitivity cardiac troponin T on survival and rehospitalization after transcatheter aortic valve replacement. Catheter Cardiovasc Interv Off J Soc Card Angiogr Interv 98(6):E881–E888 10.1002/ccd.29781 34076331

[24] Li Y, Pei H, Zhou C, Lou Y (2020) Pre-procedural elevated cardiac troponin predict risk of long-term all-cause mortality after transcatheter aortic valve replacement: a meta-analysis of prospective studies. Biomark Biochem Indic Expo Response Susceptibility Chem 25(2):164–170 10.1080/1354750X.2020.1714736 31920111

[25] Filomena D, Monosilio S, Cimino S, Maestrini V, Luongo F, Neccia M et al (2023) Prognostic role of pre- and postinterventional myocardial injury in patients undergoing transcatheter aortic valve implantation. Minerva Cardiol Angiol 71(1):77–82 33944532 10.23736/S2724-5683.21.05630-1

[26] Reents W, Barth S, Griese DP, Winkler S, Babin-Ebell J, Kerber S et al (2019) Transfemoral versus transapical transcatheter aortic valve implantation: a single-centre experience. Eur J Cardio-Thorac Surg Off J Eur Assoc Cardio-Thorac Surg 55(4):744–750 10.1093/ejcts/ezy363 30418538

[27] Biancari F, Rosato S, D’Errigo P, Ranucci M, Onorati F, Barbanti M et al (2016) Immediate and intermediate outcome after transapical versus transfemoral transcatheter aortic valve replacement. Am J Cardiol 117(2):245–251 26639038 10.1016/j.amjcard.2015.10.036

[28] Schoepfer H, Nestelberger T, Boeddinghaus J, Twerenbold R, Lopez-Ayala P, Koechlin L et al (2020) Effect of a proposed modification of the type 1 and type 2 myocardial infarction definition on incidence and prognosis. Circulation 142(21):2083–2085 33226874 10.1161/CIRCULATIONAHA.120.048920

