# Supplementary material for: The impact of myocardial injury on outcomes in TAVI patients
Source: Clin Res Cardiol. 2024 Dec 11;114(3):385–94. doi: 10.1007/s00392-024-02585-1 (PMC11913931; doi:10.1007/s00392-024-02585-1)
Supplement: Supplementary file 1 — Supplementary file1 (DOCX 366 KB) [file 392_2024_2585_MOESM1_ESM.docx]

Supplemental Figure 1: Troponin value change within 30 days pre- and 72h post-interventionally by device-access.

Central Illustration

**Supplemental Tables**

**Supplemental table 1: Comparison of characteristics between patients with and without available high-sensitivity troponin T-levels**

| **Variable** | **Overall, N = 1,480***^1^* | **No trop, N = 827***^1^* | **Trop, N = 653***^1^* | **p-value***^2^* |
| --- | --- | --- | --- | --- |
| **Basic characteristics** | | | | |
| Sex |  |  |  | 0.032 |
| Male | 748 (51%) | 396 (48%) | 352 (54%) |  |
| Female | 725 (49%) | 424 (52%) | 301 (46%) |  |
| Age | 82.7 (78.8, 86.3) | 83.0 (78.8, 86.5) | 82.5 (78.9, 85.9) | 0.3 |
| BMI | 26.2 (23.4, 29.4) | 25.9 (23.4, 29.4) | 26.4 (23.4, 29.4) | 0.3 |
| Euro SCORE II | 2.20 (1.30, 4.29) | 2.13 (1.28, 4.10) | 2.30 (1.36, 4.68) | 0.046 |
| STS calculated risk of mortality | 3.3 (2.2, 5.6) | 3.3 (2.2, 5.5) | 3.4 (2.2, 5.7) | 0.6 |
| **Preconditions** | | | | |
| Diabetes | 423 (29%) | 226 (28%) | 197 (30%) | 0.3 |
| Dyslipidemia | 865 (59%) | 461 (56%) | 404 (62%) | 0.029 |
| Hypertension | 1,194 (81%) | 651 (79%) | 543 (83%) | 0.067 |
| Peripheral artery disease | 251 (17%) | 108 (13%) | 143 (22%) | <0.001 |
| Coronary artery disease | 828 (56%) | 439 (54%) | 389 (60%) | 0.020 |
| Myocardial infarction | 250 (17%) | 134 (16%) | 116 (18%) | 0.5 |
| eGFR (Cockroft-Gault) | 51 (38, 66) | 50 (37, 65) | 52 (38, 68) | 0.2 |
| Hemoglobin | 126 (115, 137) | 126 (115, 136) | 127 (114, 138) | 0.4 |
| **Previous intereventions** | | | | |
| Any heart surgery | 145 (9.8%) | 67 (8.2%) | 78 (12%) | 0.016 |
| Surgical aortic valve replacement | 48 (33%) | 30 (45%) | 18 (23%) | 0.007 |
| Coronary artery bypass grafting | 112 (77%) | 45 (67%) | 67 (86%) | 0.007 |
| Prior pacemaker | 151 (10%) | 91 (11%) | 60 (9.2%) | 0.2 |
| Percutaneous coronary intervention | 515 (35%) | 272 (33%) | 243 (37%) | 0.11 |
| **ECG characteristics** | | | | |
| Any AV block | 219 (18%) | 119 (18%) | 100 (19%) | 0.7 |
| Right or Left bundle branch block |  |  |  | 0.4 |
| LBBB | 150 (12%) | 76 (11%) | 74 (13%) |  |
| RBBB | 152 (12%) | 88 (13%) | 64 (11%) |  |
| No | 960 (76%) | 539 (77%) | 421 (75%) |  |
| Rhythm |  |  |  | 0.7 |
| Sinus | 906 (69%) | 498 (68%) | 408 (71%) |  |
| Atrial_fibrillation | 308 (24%) | 177 (24%) | 131 (23%) |  |
| Paced_rhythm | 84 (6.4%) | 51 (7.0%) | 33 (5.7%) |  |
| Other | 12 (0.9%) | 7 (1.0%) | 5 (0.9%) |  |
| **Echocardiography parameters** | | | | |
| LVEF | 58 (45, 63) | 58 (45, 63) | 57 (45, 63) | >0.9 |
| Aortic valve mean gradient | 44 (36, 52) | 43 (36, 52) | 44 (36, 52) | 0.9 |
| Aortic valve peak gradient | 66 (48, 80) | 66 (48, 80) | 66 (47, 79) | 0.7 |
| Aortic valve area | 0.80 (0.60, 0.90) | 0.77 (0.60, 0.90) | 0.80 (0.60, 0.90) | 0.044 |
| **Intervention characteristics** | | | | |
| Implanted device type |  |  |  | 0.004 |
| balloon-expandable | 419 (29%) | 206 (25%) | 213 (33%) |  |
| self-expandable | 958 (65%) | 552 (68%) | 406 (62%) |  |
| mechanical-expandable | 91 (6.2%) | 58 (7.1%) | 33 (5.1%) |  |
| Implanted device size [mm] |  |  |  | 0.3 |
| ≤ 25 | 479 (33%) | 257 (32%) | 222 (34%) |  |
| > 25 | 987 (67%) | 558 (68%) | 429 (66%) |  |
| Access site |  |  |  | <0.001 |
| extrathoracic | 1,314 (89%) | 798 (97%) | 516 (79%) |  |
| intrathoracic | 159 (11%) | 22 (2.7%) | 137 (21%) |  |
| Implantation duration [min] | 65 (52, 80) | 62 (52, 76) | 65 (52, 85) | 0.002 |
| Hospitalization [days] | 6.1 (4.1, 9.0) | 5.1 (4.1, 8.1) | 7.0 (4.1, 9.1) | <0.001 |
| Contrast volume [ml] | 165 (132, 211) | 167 (135, 210) | 165 (129, 217) | 0.7 |
| *^1^* n (%); Median (IQR) | | | | |
| *^2^* Pearson’s Chi-squared test; Wilcoxon rank sum test; Fisher’s exact test | | | | |

**Supplemental table 2: Uni- and multivariable analysis after adjustment for age, eGFR and peri-procedural myocardial injury according to VARC3 OR VARC2**

|  | **Multivariable (adj. for age & eGFR)** | |
| --- | --- | --- |
| **Variable** | **HR (95% CI)** | **p-value** |
| **Death, 1y^1^** | | |
| PPMI (VARC3) | 1.97 (0.94 - 4.13) | 0.134 |
| PPMI (VARC2) | 1.21 (0.7 - 2.07) | 0.571 |
| **Major adverse events, 1y^1^** | | |
| PPMI (VARC3) | 3.02 (1.73 - 5.26) | <0.001 |
| PPMI (VARC2) | 0.66 (0.42 - 1.04) | 0.136 |
| **VARC-3-defined PPMI^2^** | | |
| Age | 0.95 (0.90 - 1.02) | 0.153 |
| eGFR | 0.95 (0.93 - 0.97) | <0.001 |
| ^1^Cox-regression model with and without adjustment for age and renal function | | |
| ^2^Logistic regression with penalized likelihood for low incidence events | | |

**Supplemental table 3: Multivariable analysis after adjustment for device type, access type and peri-procedural myocardial injury according to VARC3 OR VARC2**

|  | **Multivariable** | | | | |
| --- | --- | --- | --- | --- | --- |
|  | **Device type** | | **Access Site** | | |
| **Variable** | **HR (95% CI)** | **p-value** | | **HR (95% CI)** | **p-value** |
| **Death, 1y^1^** | | | | | |
| PPMI (VARC3) | 3.36 (1.44 – 7.84) | 0.005 | | 2.77 (1.16 – 6.63) | 0.024 |
| PPMI (VARC2) | 1.14 (0.59 - 2.18) | 0.700 | | 1.27 (0.65 – 2.49) | 0.485 |
| **Major adverse events, 1y^1^** | | | | | |
| PPMI (VARC3) | 4.02 (2.38 - 6.81) | <0.001 | | 3.70 (1.92 – 7.13) | <0.001 |
| PPMI (VARC2) | 0.65 (0.38 – 1.13) | 0.131 | | 0.69 (0.39 – 1.21) | 0.194 |
| **VARC-3-defined PPMI^2^** | | | | | |
| Access Site | 4.56 (1.95 - 10.82) | <0.001 | |  |  |
| Device Type | 1.08 (0.46 - 2.63) | 0.853 | |  |  |
| ^1^Cox-regression model with and without adjustment for age and renal function  ^2^Logistic regression with penalized likelihood for low incidence events | | | | | |
